# Ghana’s National Health insurance scheme and maternal and child health: a mixed methods study

**DOI:** 10.1186/s12913-015-0762-y

**Published:** 2015-03-17

**Authors:** Kavita Singh, Isaac Osei-Akoto, Frank Otchere, Sodzi Sodzi-Tettey, Clare Barrington, Carolyn Huang, Corinne Fordham, Ilene Speizer

**Affiliations:** Department of Maternal and Child Health, Gillings School of Global Public Health, University of North Carolina at Chapel Hill, Chapel Hill, NC USA; Carolina Population Center, University of North Carolina at Chapel Hill, Chapel Hill, NC USA; Institute of Statistical, Social and Economic Research, University of Ghana, Accra, Ghana; Department of Public Policy, College of Arts and Sciences, University of North Carolina at Chapel Hill, Chapel Hill, NC USA; The Institute for Healthcare Improvement (IHI), Cambridge, MA USA; Department of Health Behavior, Gillings School of Global Public Health, University of North Carolina at Chapel Hill, Chapel Hill, NC USA

**Keywords:** Antenatal care, Early care-seeking, Distance to facilities, Facility delivery, Ghana and insurance

## Abstract

**Background:**

Ghana is attracting global attention for efforts to provide health insurance to all citizens through the National Health Insurance Scheme (NHIS). With the program’s strong emphasis on maternal and child health, an expectation of the program is that members will have increased use of relevant services.

**Methods:**

This paper uses qualitative and quantitative data from a baseline assessment for the Maternal and Newborn errals Evaluation from the Northern and Central Regions to describe women’s experiences with the NHIS and to study associations between insurance and skilled facility delivery, antenatal care and early care-seeking for sick children. The assessment included a quantitative household survey (n = 1267 women), a quantitative community leader survey (n = 62), qualitative birth narratives with mothers (n = 20) and fathers (n = 18), key informant interviews with health care workers (n = 5) and focus groups (n = 3) with community leaders and stakeholders. The key independent variables for the quantitative analyses were health insurance coverage during the past three years (categorized as all three years, 1–2 years or no coverage) and health insurance during the exact time of pregnancy.

**Results:**

Quantitative findings indicate that insurance coverage during the past three years and insurance during pregnancy were associated with greater use of facility delivery but not ANC. Respondents with insurance were also significantly more likely to indicate that an illness need not be severe for them to take a sick child for care. The NHIS does appear to enable pregnant women to access services and allow caregivers to seek care early for sick children, but both the quantitative and qualitative assessments also indicated that the poor and least educated were less likely to have insurance than their wealthier and more educated counterparts. Findings from the qualitative interviews uncovered specific challenges women faced regarding registration for the NHIS and other barriers such lack of understanding of who and what services were covered for free.

**Conclusion:**

Efforts should be undertaken so all individuals understand the NHIS policy including who is eligible for free services and what services are covered. Increasing access to health insurance will enable Ghana to further improve maternal and child health outcomes.

## Background

Ghana has made considerable progress towards Millennium Development (MDG) 4 which is focused on reducing under-five mortality and MDG 5 which is focused on reducing maternal mortality. Ghana currently has a maternal mortality ratio (MMR) of 350 maternal deaths per 100,000 live births [1] and under-five mortality estimated at 78 under-five deaths per 1000 live births [2]. These estimates are declines from a MMR of 580/100,000 [1] and under-five mortality of 122/1000 in 1990 [2]. Whether or not the two-thirds reduction in under-five mortality and the three-quarters reduction in maternal mortality from 1990 to 2015 as outlined by the MDGs are met, the progress so far has been laudable. A key strategy for Ghana to make further improvements in maternal and child health is improving equitable access to health services. According to the 2011 Ghana Multi-Cluster Indicator Survey (MICS), only 37.8% of women in the lowest wealth quintile had a skilled birth attendant (SBA) at their most recent delivery compared to 97.4% of women in the richest wealth quintile [3]. Likewise according to the 2008 Ghana Demographic and Health Survey (DHS) among caregivers who had a child with a fever in the past two weeks, only 41% of caregivers in the lowest wealth quintile sought care from a health provider compared to 80% in the highest wealth quintile [4]. A study using regression-based measures and data from the 2008 DHS found pro-rich inequities in several key maternal and child health outcomes and interventions [5].

With the goal of providing health care to all by attempting to remove cost as a barrier, the government of Ghana created the National Health Insurance Scheme (NHIS) in 2003, and this program became fully operationalized in 2005. Prior to the implementation of this scheme, health care services were paid for mainly by user fees (also referred to as cash-and-carry), which research has shown is a system that disenfranchises poor and vulnerable individuals from accessing health services [6-8]. The NHIS is financed primarily through a national tax of 2.5% on goods and services and social security taxes for formal workers [9]. Individual premiums are relatively low and were about $10 USD in 2010 [10]. Insurance registration is required for all nationals de facto though there are no penalties for those who fail to enroll. Coverage is meant to be free for certain groups of vulnerable populations including the elderly (defined as individuals over 70), children under 18 and the indigent. Pregnant women and their newborns under three months were added to the exemption list in 2008 through a Maternal Health Care Program (which is described below). The NHIS covers a comprehensive range of health services and all drugs on the National Health Insurance Authority (NHIA) Medicines List. In 2010 the NHIA reported that 62% of the national population was ever enrolled in the program [10], but active current membership is about 35% of the population [11].

Before the introduction of the NHIS, the Government of Ghana implemented a policy exempting women from delivery care fees in the four poorest regions of the country - Northern, Upper East, Upper West and Central. This policy was scaled up to all regions in 2005 with the goal of giving all women free delivery care including Cesarean sections. The Ministry of Health discontinued the policy due to lack of funding during the same year the government implemented the NHIS. This created a loophole whereby women who were not enrolled in the NHIS plan would be required to pay for maternal care. As a result, the Ministry of Health implemented a new Maternal Health Care Program which exempted women from paying for their care out of pocket upon confirmation of pregnancy following enrollment into the NHIS scheme. Services covered include six antenatal care (ANC) visits, delivery care (including care for complications), two postnatal care (PNC) visits within six weeks of childbirth and care of infants up to three months of age. After three months a child would be eligible for an exemption as an individual under 18 years of age, but it would be the responsibility of the parents to register the child [12-14].

With the strong emphasis on maternal and child health, an expectation of the NHIS is that women enrolled in the program will have improved use of relevant maternal and child health services and ultimately improved health outcomes. However, only a handful of studies have delved into this issue. A study on the impact of the NHIS found increased use of facility delivery in the Brong Ahafo Region particularly among the poor [14]. Mensah et al. [15] found that NHIS enrollees in the Brong Ahafo and Upper East Regions were more likely to receive ANC and to have a facility delivery. The study also noted improved birth outcomes and reduced infant mortality among enrollees compared to non-enrollees. An earlier assessment of the pre-NHIS maternal delivery care exemption from 2004–2006 in Northern Ghana found that women who knew that delivery care was free were more likely to deliver in a health facility than those without the knowledge [13]. The lack of a measure of the precise timing of health insurance coverage has been mentioned as a limitation by both Dzakpasu et al. [14] and Mensah et al. [15]. Our data indicates that many women have coverage while pregnant due to the maternal health program, but they go off the program shortly afterwards.

The objective of this study is to describe women’s experiences with the NHIS and to assess associations between insurance and skilled facility delivery, antenatal care and early care-seeking for sick children. Our study adds to the limited literature on this topic by using a mixed-methods approach to examine both maternal and child health outcomes and by including precise measures of consistency of health insurance coverage and coverage during the exact time of pregnancy together with in-depth narratives about insurance experiences. We also explore whether there are differences between poverty level and education among those insured and not insured within our particular sample, thus adding to the research aiming to understand whether the NHIS is meeting its intention of reaching the poor [16-21].

## Methods

### Overall study design and setting

Data came from a mixed-methods baseline assessment for an evaluation of the Maternal and Newborn Referrals Project. The project is being implemented by the Institute for Healthcare Improvement (IHI), the National Catholic Health Service (NCHS) and the Ghana Health Service (GHS). Fieldwork for the evaluation’s baseline assessment was conducted between May and June 2012 in the Northern and Central Regions of Ghana.

We used a simultaneous approach whereby we collected and analyzed quantitative and qualitative data at the same time and then integrated our findings related to the study aims of this paper [22]. Such an approach enabled us to obtain a richer and more comprehensive understanding of individual behaviors and community and contextual dynamics related to insurance, than a single method alone [23]. Mixed-methods approaches are increasingly being used in the study of diverse models of health insurance across Africa, reflecting the need to integrate multiple perspectives and sources of information to understand the complex determinants of insurance uptake and the relationships between insurance and outcomes [21,24,25].

Information from the baseline assessment was used to inform interventions under the Maternal and Newborns Referrals Project. Ethics review approval for the study was obtained by the University of North Carolina at Chapel Hill and the GHS. Informed consent was obtained from all study participants.

### Description of the quantitative component

#### Sampling and fieldwork

The quantitative assessment included a household survey with 1267 women and interviews with 62 community leaders, which is about one leader per community. (In two large communities two leaders were interviewed.) The purpose of the household survey was to obtain information on knowledge, attitudes and practices regarding maternal and child health services. The focus of the community leader questionnaire was to understand community-level factors and barriers to the use of health services.

The household survey employed the 30 by N cluster sample design, which is commonly used in child survival programs [26]. The overall sampling strategy was designed to meet the evaluation objectives for the Maternal and Newborn Referral Project. At baseline, the goal was to include a large sample of recently pregnant women to identify their experiences with pregnancy, childbirth, and newborn health. Thus, we started with a sampling strategy of a 30 by 7 approach to identify thirty clusters per region (Northern or Central), and seven recently pregnant women (pregnant in the last 12 months) in each cluster were to be randomly selected for interview (see below). To supplement the sample of 210 recently pregnant women, we also included 14 nearby neighbor women (ages 15–49) who were not necessarily recently pregnant to permit an examination of maternal and newborn health knowledge, attitudes, and behaviors of women in the community. The target sample was 630 women (210 women with a recent birth and 420 additional women) in both the Northern and Central Regions. (The target sample size was actually exceeded by seven for a total of 1267 instead of 1260 women).

Thirty randomly selected communities within three districts in the Northern Region and 30 randomly selected communities in three districts of the Central Region were included in the sample. The districts were chosen by the project implementation team based on current and planned project activities. The recently pregnant women were randomly sampled from a list of all recently pregnant women in the community (determined through interviews with community leaders and health workers). Cluster sampling is advantageous because it provides a means to obtain a representative sample from the region without undertaking a census of households in the community. In this case, based on an exhaustive list of communities in the six districts (three in Northern region and three in Central region), it was possible to select a random sample of communities to represent the study districts. This is an efficient sampling method, but it leads to biased standard errors due to the correlation between observations from the same cluster. We explain our approach for accounting for the biased standard errors in the quantitative analysis section.

The target sample size of 1260 was determined based on the broader objectives of the evaluation study of looking at changes in key outcomes over time. For the purpose of our descriptive paper which used baseline data only and accounting for plausible design effect, our sample size is adequate to obtain precise estimates of our key outcomes.

#### Quantitative outcome variables

Two maternal outcomes, facility delivery by a SBA and four or more ANC visits were studied for a woman’s most recent pregnancy in the last three years. One child health outcome, health seeking by severity of illness for children under-five, was also included in the analysis. The World Health Organization (WHO) defines a SBA as an individual trained to proficiency in the skills needed to manage normal pregnancy, childbirth and the immediate postnatal period, and in the identification, management and referral of complications in women and newborns [27]. Increasing the percent of deliveries attended by a SBA in a health facility is widely regarded as a key strategy to reduce maternal mortality [28]. The World Health Organization (WHO) promotes at least four ANC visits as a strategy to improve both maternal health and birth outcome [29]. ANC is also a means to link women to health services, and women who attend ANC visits have been shown to be more likely to have a facility delivery [30]. The child health outcome was defined as a binary variable indicating a women’s perspective on whether or not an illness had to be severe (either very serious or somewhat serious versus slightly serious or non-serious) for her to bring her under-five child to a health facility. Early care-seeking is an important strategy to reduce under-five mortality because illnesses such as malaria and pneumonia can progress rapidly in young children if treatment is delayed. It is estimated that poor or delayed care-seeking contributes to up to 70% of under-five deaths [31].

#### Quantitative key independent variables

##### Health insurance

Two health insurance variables were studied – NHIS coverage in the past three years and coverage while pregnant. In the quantitative household questionnaire a matrix was used to ask women about NHIS coverage currently and during the past seven years, but only data on current coverage and coverage during the past three years was used for this variable because of missing responses beyond three years. A decision was made to make the coverage in the past three years variable categorical because conceptually we expected differences between women with coverage for all three years versus women with no coverage and women with coverage for 1–2 years (which often reflected the calendar year(s) that they were pregnant). The coverage variable was categorized as all three years, one to two years and no coverage and was included in the analysis for all three outcomes. The NHIS coverage while pregnant variable was binary and was included in the analyses for the maternal health outcomes for women with a pregnancy in the past three years. We expected both health insurance variable be associated with increased use of services.

##### Community-level factors

Distance and transportation (either the availability of transport or money for transport) were noted as barriers to maternal and child health services in this study population and other populations in sub-Saharan Africa [32-37]. Staffing of health facilities and shortages of health workers remain a challenge for many rural areas of low and middle income countries [38]. Within Ghana the Northern region is a particular concern and has the lowest doctor-to-population and nurse-to-population ratios [39]. Though Ghana health policy indicates that health centers should be staffed by a midwife, many health centers in the Northern region face gaps in coverage. To capture these community-level factors, community leaders were asked questions about access (distance to a health facility) and availability of health services (whether midwives are present at the nearest health facility). Two distance variables were created: 1) distance to the nearest health center or hospital used in the analysis of maternal health outcomes, and 2) distance to the nearest health post, health center or hospital used in the analysis of the child health outcome. Separate variables were created because the most common source of ANC and skilled delivery are health centers and hospitals that are staffed by midwives. Conversely, for the child health outcomes families have a broader choice of health care options to meet the needs of sick children including health posts, health centers, and hospitals. The midwife staffing variable was included in the analyses for both the maternal health outcomes; it is measured based on the presence of a midwife at the nearest facility (categorized as throughout the year, part of the year, not at all or don’t know). We expect that distance and lack of a midwife at the nearest facility will be deterrents to care-seeking.

##### Other independent variables

Several individual level control variables were included in the analysis based on previous studies that have demonstrated that these variables are associated with the maternal and child health outcomes of interest [32-37,40-43]. These control variables include the woman’s age, parity at the time of last birth, education level, religion, working status, urban/rural residence and ethnicity (dominant for the community or not dominant). A variable indicating region – Northern or Central was also included. Age of child (categorized as <1 year, 1–2 years and 3–4 years) was included in the analysis for the early care-seeking outcome. A household-level wealth variable was included and was constructed by first examining characteristics of households which distinguished poor and non-poor households in the 2008 Ghana DHS and characteristics which were also included in the Maternal and Newborn Referrals Survey. Type of toilet, source of drinking water, and cooking fuel were the characteristics that best differentiated the lowest and highest wealth quintiles in the Ghana DHS. These variables were then used to create a wealth variable distinguishing households that had all characteristics as the wealthiest, one or two as middle income and zero as the poorest. A similar approach applying three characteristics to classify poor and non-poor households has been used previously in studies in sub-Saharan Africa [44,45]. As a check, our wealth variable was examined relative to the number of assets (i.e. television, radio and other household items) and was found to be consistent in the Northern and Central Regions such that poor women had the least number of household assets compared to wealthier women.

### Description of the qualitative component

#### Sampling and fieldwork

The study included qualitative interviews including birth narratives with 20 mothers, 18 fathers, in-depth interviews with 5 health care providers and 3 focus groups with community leaders and key informants. The purpose of the birth narratives with mothers and fathers was to elicit their personal experiences with complications during pregnancy and delivery and to understand the role of referral systems. The in-depth interviews and focus groups aimed to obtain the perceptions, opinions and norms of health care providers and community leaders regarding barriers to effective referrals, community context and strategies for improvement.

The field team asked health workers at health centers in the two study regions to generate a list of approximately forty women and/or their newborns who experienced complications prior to or following their arrival at a health facility during pregnancy or delivery in the past year. With the assistance of a community health worker or assemblyman, the team requested interviews with twenty mothers and twenty fathers on this list. One health facility was selected in both the Northern and Central Regions, and two to three health workers were recruited to participate. For the focus groups, community assemblymen and other community mobilizers recruited community health volunteers, traditional birth attendants (TBAs), chemical sellers, transport workers, and community leaders.

#### Qualitative interview guides

Qualitative interviews and focus groups were conducted using semi-structured interview guides that included questions and probes related to the assessment aims but also allowed for flexibility. Birth narrative interviews with mothers and fathers elicited in-depth descriptions of experiences with complications and referrals in an attempt to situate these experiences in the broader context. Interviews with health providers elicited their experiences and opinions about referrals. Focus groups with community leaders enabled an understanding of community-level norms and attitudes around maternal and child health. Across all interviews key topics included family and community dynamics, socio-cultural beliefs, structural barriers and facilitators (i.e. transport), and quality of care. While some participants discussed post-natal complications, the majority of interviews focused on antenatal and delivery experiences.

### Analysis

#### Quantitative analysis

There were two components to the quantitative analysis - 1) bivariate logistic regression and multivariable logistic regression to understand the influence of health insurance on the outcomes of interest after controlling for individual, household and community factors and 2) chi-square analyses to understand differences between those who had and did not have insurance by wealth and educational status. As explained earlier the variables for the multivariable analysis were selected based on theory and prior research. Because of the cluster sampling methodology, the analysis accounts from cluster level variation in the outcome variables. This was done by presenting robust estimates of variance.

#### Qualitative analysis

For the qualitative analysis, all interviews were audio-recorded, transcribed verbatim and translated. We used an inductive approach to the qualitative analysis in which we initiated the analysis without *apriori* hypotheses and we constructed our interpretation based on the salient themes we identified in the participant’s narratives related to their insurance experiences [46]. We prepared analytic summaries for all individual and group interviews to capture the main story of the birth complication or key themes related to ANC, skilled delivery and the referral system [47]. Based on these summaries, we developed a coding scheme that we systematically applied to all transcripts using the Atlas.ti software including codes related to insurance coverage, insurance registration and use of insurance during referral and delivery experiences [48]. We then developed analytic matrices based on the outputs of the coding to summarize findings for each key theme and compare between different categories of participants [49].

## Results

### Descriptive quantitative results: independent variables

Descriptive results for the quantitative sample of women with a child under-five are contained in Table 1. About 88% of women in the sample were currently married and about half had no formal education and were unemployed or doing unpaid work. In terms of wealth 40% of women were classified as poor, 43% as in the middle and 17% as wealthy. About 78% of the sample was part of the dominant ethnicity group for their particular region while 22% were of a minority ethnicity. Fifty-seven percent of the sampled women were Christian and 43% were Muslim. The majority lived in rural areas (77%).

**Table 1:** **Description of the Sample of Women with a Child Under-five (N = 969)**

**Characteristic**		**Percentage**
Women’s age (years)	15-19	7.5
	20-24	25.4
	25-34	44.9
	35-49	22.2
Parity	0-1	22.0
	2-3	30.8
	≥4	47.2
Current marital status	Married	87.8
	Not Married	12.2
Education	None	51.1
	Primary	19.6
	Secondary or more	29.3
Working status	Unemployed/unpaid	51.5
	Self-employed	44.2
	Paid	4.3
Wealth category	Poorest	40.3
	Middle	42.5
	Richest	17.2
Religion	Christian	56.7
	Muslim	43.3
Belongs to the dominant ethnicity for region	Yes	77.9
	No	22.1
Locality of residence	Urban	23.1
	Rural	76.9
Region of residence	Northern	49.7
	Central	50.3
Child’s age	Under 1	43.0
	1-3	20.9
	3 to 5	36.1

The key quantitative independent variables are presented in Table 2. In terms of insurance coverage over the past three years 19.2% of the sample had insurance for all three years, 67.8% had coverage for one or two years and 13.0% had no coverage. Ninety-three percent of women with a pregnancy in the past three years had insurance during the exact timing of their pregnancy. Fifty-eight percent of respondents were within one kilometer (km) of a health center or hospital, while 26% were six km or more away. When health posts were included in the distance measure, 66% of communities were less than one km away and 10% were six km or more away. Forty-nine percent of communities had a midwife present at their nearest facility for the entire year, while 33% indicated their nearest facility was not staffed by a midwife at all.

**Table 2:** **Description of the health insurance and community variables for the aample of women with a child under-five (N = 969)**

**Characteristic**		**Percentage**
Health insurance coverage	3 Years	19.2
	1-2 Years	67.8
	No Coverage	13.0
Health insurance while pregnant	Yes	92.9
	No	7.1
Midwife presence at nearest facility	Whole Year	49.1
	Part of Year	9.7
	Not at all	33.3
	Don’t Know	7.8
Distance to nearest health center/hospital	Less than 1 km	58.1
	1 km-5 km	17.1
	6 km or more	24.9
Distance to nearest health post/health center or hospital	Less than 1 km	66.3
	1 km-5 km	23.8
	6 km or more	9.9

### Qualitative results-insurance

Most of the participants in the qualitative interviews also indicated that they (or their wives) had insurance during pregnancy. While we did not systematically ask all mothers and fathers about their insurance status, most mentioned getting registered in response to the woman’s pregnancy or already having insurance when they became pregnant. They used the word “snapped” to describe the registration process, which refers to getting their picture taken during registration, in addition to submitting the required paperwork and fee. Most mothers and fathers described positive and fairly streamlined experiences with the insurance registration process, with some even describing that health workers came to their communities to sign them up. There did appear to be some inconsistencies regarding whether there was a cost to registration with some participants describing paying a fee and others describing health workers coming to their communities to sign them up free of charge. Several mothers and fathers described being charged money to get enrolled when they thought it should be free, having issues with expired insurance, being told to come back to the clinic several times before they could be registered, requiring the father to be present in order to enroll the woman, and/or simply never receiving the insurance card. One woman described still dealing with her insurance coverage issues at the time of her delivery, as she negotiated the extreme pain of labor. The quote below from a 17-year-old mother from the Northern region with an 8th grade education reflects some of these registration issues,

…when I got pregnant, I didn’t bring my health insurance to this town and I went to snap and they took our money. Eeh, when we went they said we and our husbands are going to snap and we asked them [because] they used to snap free, free for pregnant women and they said no, that now they don’t snap like that. That they now snap you and your husband and they will snap you and take…and take you money and we asked them how much was it and they said GHC 12.20 [about $6 US] and we paid and snapped.

This quote also reflects a phenomenon called double registration, which occurs when women move locations during the three month waiting period following registration and then re-register in their new location [50].

Beyond the registration process, most mothers and fathers identified not having to pay for antenatal care and delivery services as the main benefit of insurance. One 38-year-old father from the Central region with no education described that due to insurance, his family did not experience excessive costs related to his wife’s delivery in a facility,

Well, I think that because the health insurance was catering for all the cost that one would have incurred, nothing indeed prevented her or any other person from going to the hospital during such times. Moreover, the hospital is located just here. Thus, it was not too far from here so she did her best to go at any time that she needed to do so.

In addition to the financial coverage, this quote also highlights the importance of location for a facility based delivery as distance can create elevated costs, above and beyond the coverage of insurance. Beyond this financial benefit, the 17-year-old woman quoted above attributed her insurance as facilitating efficient antenatal care,

Ok, since I had the health insurance, it used to help me because when I use to take it to the hospital it, I did not have struggles. When I just go and show the health insurance to them, I see direct and get my care.

### Descriptive quantitative data – outcomes

**Figure 1:**
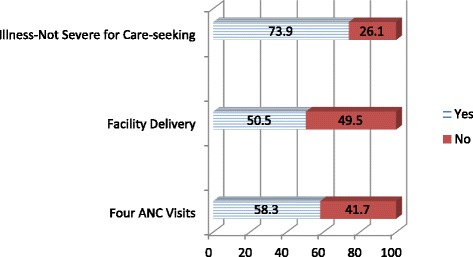
**Outcome variables.**

Descriptive data for the quantitative outcome variables are presented in Figure 1. Fifty-eight percent of women pregnant in the last three years or who were currently about nine months pregnant had at least four ANC visits, and 51% of women with a live birth in the past three years delivered in a health facility. Among women with a child under-five, 74% indicated that an illness need not be severe for them to take their child to a health facility.

### Quantitative results - bivariate analyses

Table 3 contains the results of the bivariate analyses of the outcomes of interest with the health insurance variables. Having health insurance over the past three years was significantly associated with delivery with a skilled birth attendant in a health facility. Odds ratios were 2.9 (CI: 2.0-4.4; p < 0.001) for insurance for one to two years and 2.5 (CI: 1.4-4.2; p < 0.001) for insurance for all three years compared to no coverage. Having insurance at the exact time of pregnancy was also significantly associated with a facility delivery (OR: 2.5; CI: 1.5-4.1; p < 0.001). Insurance for three years compared to no coverage of health insurance was significantly associated with four or more ANC visits (OR = 1.8; CI: 1.1-3.1; p < 0.05), but there was no association between the insurance coverage while pregnant variable and the ANC outcome. Caregivers with some insurance over the past three years were more likely to say that an illness need not be severe for them to take their child to a health facility. Odds ratios were 1.8 (CI: 1.2-2.5; p < 0.05) for one or two years of insurance coverage and 2.0 (CI: 1.2-3.3; p < 0.001) for coverage for all three years compared to no coverage.

**Table 3:** **Bivariate analysis between health insurance coverage and outcomes**

	**Facility delivery (N = 788)**	**Four or more ANC visits (N = 767)**	**Illness need not be severe for care-seeking (N = 955)**
Health insurance coverage			
No coverage	Ref	Ref	Ref
1-2 years	2.9***(1.96-4.39)	1.2 (0.80-1.70)	1.7***(1.24-2.52)
3 years	2.5** (1.64-4.19)	1.8* (1.06-3.13)	2.0*(1.16-3.31)
Health insurance while pregnant			
No	Ref	Ref	
Yes	2.5**(1.45-4.14)	1.5 (0.86-2.58)	NA

Note: N’s presented are the analysis sample; overall, 50.5% of women had a facility delivery, 58.3% of women had four or more ANC visits, and 73.9% reported that an illness need not be severe to seek care.

*p < 0.05 **p < 0.01 ***p < 0.001.

### Quantitative results - multivariable analyses

Results from the full multivariable regression models are in Table 4. Having insurance during the exact time of pregnancy was significantly associated with facility delivery (OR = 2.5; CI: 1.3-4.5; p < 0.01) but not with ANC visits. Women who had some insurance coverage over the past three years were more likely to have a facility delivery, though the association was only significant for coverage for one to two years compared to no coverage (OR = 1.8; CI:1.1-3.0; p < 0.05). Caregivers with some insurance coverage were significantly more likely to say they would take a child with a non-severe illness to a health facility than those with no insurance over the past three years. Odds ratios were 1.7 (CI: 1.1-2.4; p < 0.01) for one or two years of coverage and 2.0 (CI: 1.1-3.4; p < 0.05) for all three years compared to no coverage. Greater distances from the nearest health facility were significantly and negatively associated with the odds of a facility delivery and having four or more ANC visits. The wealthiest and most educated women were significantly more likely to have a facility delivery than their poorer and less educated counterparts. Unexpectedly, poorer women were more likely to have four or more ANC visits compared to wealthier women. Interactions between insurance and poverty were tested but found not to be significant.

**Table 4:** **Multivariable logistic regression analyses**

	**Facility delivery (N = 788)**	**Four or more ANC visits (N = 767)**	**Illness need not be severe for care-seeking (N = 955)**
Education			
None	Ref	Ref	Ref
Primary	1.3 (0.84- 2.09)	1.3(0.73-2.27)	1.6(0.90-2.75)
Secondary	2.4*** (1.52-3.92)	1.6 + (0.95-2.64)	1.4(0.79-2.37)
Wealth			
Lowest	Ref	Ref	Ref
Middle	1.8 (0.74-1.57)	0.6 + (0.38-1.03)	2.0**(1.31-2.98)
Richest	1.6*(1.04-2.47)	0.46*(0.24-0.88)	1.0(0.56-1.86)
Health insurance coverage			
No coverage	Ref	Ref	Ref
1-2 years	1.8*(1.13-2.96)	1.0(0.70-1.59)	1.7** (1.15-2.38)
3 years	1.1(0.59-2.11)	1.6(0.74-3.62)	1.96*(1.13-3.40)
Health insurance while pregnant			NA
No	Ref	Ref	
Yes	2.5**(1.35-4.46)	1.2(0.70-2.07)	
Distance to nearest health			
Center/hospital			NA
<1 km	Ref	Ref	
1 km-5 km	0.6 (0.27-1.32)	0.9(0.39-2.06)	
6 km+	0.5 + (0.23-1.02)	0.4 + (0.18-1.13)	
Distance to nearest health			
Post/health center/hospital	NA	NA	
<1 km			Ref
1 km-5 km			2.0(0.89-4.67)
6 km+			1.8(0.61-5.04)
Midwife presence at nearest facility			NA
Whole year	Ref	Ref	
Part of the year	0.4 (0.09-2.05)	0.7(0.33-1.64)	
Not at all	0.9 (0.49-1.72)	1.1(0.64-1.87)	
Don’t know	0.8 (0.22-3.13)	0.5(0.73-2.81)	
Pseudo R^2^	0.24	0.07	0.11

+ p < 0.10 *p < 0.05 **p < 0.01 ***p < 0.001.

Note: Models also control for mother’s age, parity, marital status, working status, ethnicity, residence, region and child’s age (for the early care-seeking outcome). N’s presented are the analysis sample; overall, 50.5% of women had a facility delivery, 58.3% of women had four or more ANC visits, and 73.9% reported that an illness need not be severe to seek care.

### Quantitative results – who has insurance?

**Figure 2:**
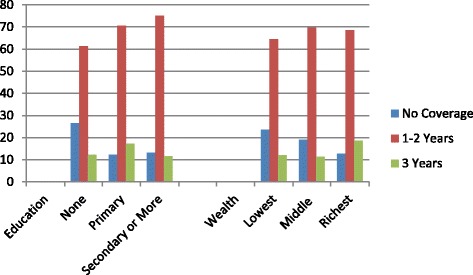
**Health insurance coverage by education (** ***X*** ^**2**^ **= 40.3; df = 4; p < 0.000) and wealth (** ***X*** ^**2**^ **= 17.0; df = 4; p < 0.002).**

Figure 2 presents the health insurance coverage variable by poverty and education level. Wealthier and more educated individuals were more likely to have some insurance coverage over the past three years than women who are poor and have no education. No differences were found for the coverage while pregnant variable. (These results are not shown).

### Qualitative results – who has insurance and barriers to having insurance

Qualitative data provide additional insights into the relationship between poverty, insurance and use of services. In the qualitative interviews with health workers and community leaders, the role of poverty and fear of referrals were identified as lingering barriers to facility-based delivery even with the NHIS system in place. As articulated by one health worker from the Central region in response to barriers to uptake of referrals,

Especially finance. Even if the person is having health insurance, you will tell the person that oh mama or papa, we have given you first aid and thing is not going so you will have to go and the person will tell you that I don’t have anything. I have to go for a loan first and I don’t know about anyone who [will] give me. Then it becomes a problem.

Others indicated that while the NHIS had addressed many of the costs barriers at the facility level, many mothers and fathers, especially those with limited resources, feared “uncertainties” related to the “unknown environment” of referral facilities as well as fear of how they would be treated. In response a health worker from the Northern Region explained how she accompanies women who are referred,

Uncertainties. I realized that they always feel that being referred there means that there will be hassles there. Unknown environment, they don’t know anybody there and for that matter, they will tell you that, I prefer to die here. That is why we have also made it a policy to go with them.

Additionally, low education levels were identified by a 34-year-old Catholic priest in a community focus group in the Northern region as exacerbating misunderstanding of the system and fueling problems related to expiration of insurance as people were not able to read and understand the policy,

And also the next thing is that some of them as he said the illiteracy rate is so high they don’t, some of them expired um-aah, it is only when there is the need to know they have a card but when they remove it maybe it has not been renewed for the past two years.

In response to the barrier of poverty, one male community leader in a focus group suggested targeting women to get registered during a harvest season, a time when farmers have more money, to address poverty as a barrier to insurance registration.

We must also encourage the women to partake in the health insurance. Some of the pregnant women may like to go to the hospital but because they do not have the health insurance or the money to pay, they stay back. But if they are encouraged and they register and renew with the health insurance, they can access these facilities for their own good. We can actually time and encourage them during cocoa season to register and renew their health insurance.

In addition to fears of referral (due to unknown costs, transport and new facilities) for pregnant women, lack of insurance and cost were identified by a health worker as determinants of delayed care-seeking for children,

But the main major problem is financial. Like for some of them you see a sick baby and you find out that the child has been sick for so many days and they will stay home giving the baby herbs before maybe somebody will see and say that I will assist so let’s go. If someone falls sick and the person does not have insurance, the person refuses to go.

This was confirmed by a 49-year-old mother from Northern region with no education who explained that she did not have insurance for her children, “… because of poverty, there is no money and the children are many. If you want to add them to enroll, there is no money.” The prior two quotes also highlight misunderstandings about the NHIS as children under eighteen are eligible for free coverage.

### Summary of results

Taken together, the quantitative and qualitative findings highlight how the NHIS has helped to address the barrier of cost, but factors such as transport, fear of unknown facilities, expenses not covered or believed to be not covered by insurance and extreme poverty served as ongoing barriers to the use of services for both mothers and their children.

## Discussion

Ghana has received considerable attention because it is one of the few countries in Africa aiming to provide universal health insurance. Ghana is also noted for implementing a single coverage program, for the strategies of raising revenue for the program and for the comprehensive coverage provided by the NHIS [18]. With the program’s specific emphasis on maternal and child health, an expectation is that enrollees would have better use of services and ultimately better maternal and child health outcomes. Our qualitative interviews suggest that most challenges with insurance from the perspective of mothers and fathers occurred during the registration process but for those who were able to “snap”, or register and receive their card, the coverage was efficient and comprehensive.

This paper adds to the limited evidence that NHIS members use more maternal health services than non-members [14,15]. After controlling for key individual, household and community level factors, this study found that individuals who had coverage during the exact timing of pregnancy were significantly more likely to have a facility delivery. Having full coverage over the past three years, however, was not significant. This may indicate that for this particular outcome what is most crucial is having insurance during the period of the actual pregnancy. There was also evidence of increased use of early care-seeking for sick children based on caregiver’s reports of when to obtain services for a sick child; this has not been studied before. Though there were positive associations between both insurance variables and four or more ANC visits, these results did not attain statistical significance. Our qualitative results yield insight into the reasons behind this lack of significance. ANC visits are generally much less expensive than a facility delivery. Indirect costs may be lower as women are often able to walk to the nearest facility for an ANC visit, but often are unable to do so while in labor. In addition based on our qualitative findings, the lack of significant associations between insurance and four or more ANC visits could also be attributed to delays in the registration process related to confusion about the requirements for registration, bureaucratic delays, and internal migration leading to issues with double registration. Thus it is important for policymakers in Ghana to facilitate the registration process for pregnant women so that they may access a sufficient number of ANC visits.

Results from this paper indicate that while the majority of women have insurance while pregnant only 19% of women in the full sample had NHIS coverage during the past three years. Explaining the benefits of insurance to individuals with children may be an important means to increase overall insurance enrollment. Several studies have delved into the issue of whether the NHIS reaches the poor and non-poor equally [16-20] with a general finding that the NHIS should do more to reach its intention of covering poor individuals. Our findings support findings from these studies. Fortunately, however, recent data does indicate that improvements are being made in equitable coverage [51]. Our qualitative findings are unique in presenting barriers faced by mothers and fathers in terms of registering for insurance. A key barrier is the lack of understanding of the policy (particularly among individuals with low literacy) including who is covered and the need for renewal of insurance. This barrier can be addressed with appropriate community-level interventions.

There are several limitations to this analysis. The distance to the nearest health facility variable assumes that a woman would go to the nearest health center or hospital for ANC and delivery care, and that she would go to the nearest health post, health center or hospital to seek care for her children. In reality some women may “bypass” the nearest facilities and chose one that it is farther away but perhaps which is perceived to be of better quality [52]. This occurrence was described in our qualitative interviews and seemed to be more of an issue for facility delivery than with antenatal care and child health care. If women are bypassing the closest facility because of concerns of quality, this may mean that the effect of our distance variable on facility delivery is attenuated as it does not represent the choice of facility that women are actually making. Recall bias could be an issue with the health insurance coverage over the past three years variable, though this is a relatively short period of time. Women may recollect well that they were covered by health insurance during their pregnancy but not be as certain about the full three year period; this would result in a stronger effect of the health insurance in pregnancy variable than the three year coverage variable. The child health outcome asks caregivers generally about how severe an illness would need to be for them to take their child to a health facility, rather than asking about actual behavior. We have also not explored why some women (8%) did not sign up for the NHIS during pregnancy even though they would not be charged. Thus our quantitative analysis may suffer from endogeneity in that there may be some unobservable differences between women who signed up for insurance and those that did not and this could lead to an over-estimation of the effect of insurance coverage. Despite the limitations, this mixed-methods analysis adds to the limited literature on the influence of the NHIS on maternal and child health outcomes.

## Conclusion

The main findings from this paper indicate that among those who are covered by the NHIS, there is greater use of skilled deliver and early care-seeking for sick children after controlling for relevant individual, household and community-level factors. The government of Ghana should continue to increase overall coverage for pregnant women and mothers of young children, particularly among the poor and least educated who we found to have lower overall coverage. In addition efforts should be undertaken to ensure that all individuals understand the NHIS policy including what is covered and who is eligible for free coverage. Community-level interventions aiming to carefully describe the policy and procedures for registration are needed to increase insurance enrollment. The process of registration for insurance should also be clear, timely and easy to follow. Increased access to insurance and thus health care can assist Ghana in coming closer to reaching MDGs 4 and 5.
